# Pterygoid bone malformation and its limitations on the effectiveness of brachycephalic airway corrective surgery in brachycephalic dogs

**DOI:** 10.1111/jsap.70028

**Published:** 2025-09-11

**Authors:** S. L. Yuen, M. A. Genain, J. F. Ladlow, N.‐C. Liu

**Affiliations:** ^1^ Department of Veterinary Medicine University of Cambridge Cambridge UK; ^2^ Sprinz & Nash Veterinary Surgeons Thame UK; ^3^ Diagnostic Imaging, Department of Veterinary Medicine, Queen's Veterinary School Hospital University of Cambridge Cambridge UK; ^4^ Granta Veterinary Specialists Cambridge UK; ^5^ School of Veterinary Medicine National Taiwan University Taipei City Taiwan; ^6^ Small Animal ENT Head and Neck Surgery National Taiwan University Veterinary Hospital Taipei City Taiwan

## Abstract

**Objectives:**

This study aimed to examine the association between pterygoid bone medialisation and treatment outcomes after upper airway surgery in three brachycephalic breeds.

**Materials and Methods:**

Dogs that underwent CT of the head followed by routine surgery for brachycephalic obstructive airway syndrome were recruited in this study. Measurements obtained from the CT images included the width of the basisphenoid bone, interpterygoid distance and cross‐sectional area of the nasopharynx. A ratio of width of the basisphenoid bone to interpterygoid distance allowed quantification of pterygoid bone medialisation. Pearson's correlations were calculated to assess the relationship between width of the basisphenoid bone: interpterygoid distance and cross‐sectional area of the nasopharynx. Additionally, width of the basisphenoid bone: interpterygoid distance was compared across breeds, as well as between dogs with good and poor surgical outcomes (post‐operative brachycephalic obstructive airway syndrome index ≥50%).

**Results:**

One hundred and forty‐four brachycephalic dogs (47 Pugs, 64 French Bulldogs and 33 Bulldogs) and 30 non‐brachycephalic controls were included in the analysis. The width of the basisphenoid bone: interpterygoid distance ratio in brachycephalic dogs was significantly higher (1.982 ± 0.379) than that of controls (1.646 ± 0.239, P < 0.001). A negative correlation was observed between width of the basisphenoid bone: interpterygoid distance and cross‐sectional area of the nasopharynx in Pugs (ρ = −0.29, P = 0.048), French Bulldogs (ρ = −0.47, P < 0.001), Bulldogs (ρ = −0.71, P < 0.001) and controls (ρ = −0.55, P = 0.002). French Bulldogs with poor surgical outcomes exhibited a significantly higher width of the basisphenoid bone: interpterygoid distance (2.366 ± 0.327) than those with good surgical outcomes (1.813 ± 0.271, P < 0.0001).

**Clinical Significance:**

Pterygoid bone medialisation is associated with nasopharyngeal narrowing, which limits the effectiveness of surgical interventions in brachycephalic obstructive airway syndrome in affected French Bulldogs. As there are no surgical options currently reported to address this condition, these findings are important in guiding clinicians in providing prognostic information to owners during elective brachycephalic obstructive airway syndrome surgery.

## INTRODUCTION

Brachycephalic obstructive airway syndrome (BOAS) is a chronic and potentially life‐threatening disease characterised by multiple obstructive airway lesions that compromise both respiration and thermoregulation. These traits are prevalent in a large population of brachycephalic dogs. These breeds that were selectively bred for their shortened snouts have been known for their strong bite and endearing appearance. Due to this selection, soft tissues within the skull do not decrease proportionally and result in a narrowed upper respiratory tract (Ekenstedt et al., 2020; Krainer & Dupré, 2022).

Common anatomical deformities associated with BOAS include stenotic nares, aberrant nasal turbinates, an elongated and thickened soft palate, macroglossia, everted palatine tonsils, and laryngeal and tracheal collapse. The excessive soft tissues surrounding the nasopharynx result in partial or complete occlusion (Ekenstedt et al., 2020; Krainer & Dupré, 2022; Song et al., 2023). This is known to be contributed by a thickened soft palate, oedema and hyperplasia of the nasopharyngeal mucosae and a relatively large tongue. Various studies utilising CT have described the characteristics of nasopharyngeal dimensions, such as the location of maximal occlusion and the positive correlation between thicker soft palates and more severe clinical presentations (Grand & Bureau, 2011; Heidenreich et al., 2016; Jones et al., 2020). Numerous surgical techniques (e.g. folded flap palatoplasty) have been developed and demonstrated to effectively alleviate this conformational obstruction by reducing the length and/or thickness of the palate (Krainer & Dupré, 2022; Liu et al., 2017; Sarran et al., 2018; Seneviratne et al., 2020). While the dorsoventral narrowing of the nasopharynx can be extensively addressed surgically, the latero‐lateral narrowing of the nasopharynx has rarely been described in the veterinary literature.

Different conformation of the pterygoid bones compared with normocephalic dogs was found in nearly 80% of French Bulldogs in a study with a small cohort (Del Mar Bovis et al., 2024). This cranial region of the nasopharynx is laterally bordered by the pterygoid bones and dorsally by the basisphenoid bone, resulting in a paucity of the pharyngeal dilator muscles compared with its caudal counterpart. Considering the limited flexibility of the airway lumen at this level and the lack of surgical options for addressing pterygoid abnormalities, such anatomical variations may contribute to increased upper airway resistance. However, further research is required to elucidate the significance of this variation in the severity and prognosis of BOAS.

The objectives of this CT based retrospective study were to (i) determine the relationship between pterygoid bone medialisation and nasopharyngeal narrowing in French Bulldogs, Bulldogs and Pugs, compared with non‐brachycephalic controls, and to (ii) assess the association between the pterygoid bone medialisation and surgical outcomes using the BOAS index. We hypothesised that pterygoid bone medialisation is a prominent feature in brachycephalic breeds, which leads to a decreased cross‐sectional area of the nasopharynx, thus serving as a negative prognostic factor for the outcomes of BOAS corrective surgery.

## MATERIALS AND METHODS

### Study subjects

French Bulldogs, Pugs and English Bulldogs referred to the Queens Veterinary Hospital (QVSH), University of Cambridge, for BOAS assessment and surgery between 2015 and 2021 were recruited into this study. The inclusion criteria were the availability of head and neck CT scans and the complete clinical history pertaining to BOAS assessment prior to surgery (history, BOAS index pre‐ and post‐operatively). The control dogs were non‐brachycephalic patients treated at the QVSH for other medical conditions and underwent CT scanning of the head and neck. Patients with lower airway diseases or head and neck abnormalities unrelated to BOAS lesions, as diagnosed via x‐rays or CT, were excluded from this study.

This study was carried out under the informed ethical consent of CR505 from the Department of Veterinary Medicine, University of Cambridge.

### Measures of BOAS severity pre‐ and post‐surgery

The BOAS severity was determined by a BOAS index, a numerical scale from 0% to 100% calculated from the respiratory parameters obtained from whole‐body barometric plethysmography as well as the BOAS respiratory functional grading system (Grades 0 to III) (Liu et al., 2015). A BOAS index of 100% denotes severely BOAS‐affected patients and 0% being BOAS free. All subjects in this study had a pre‐operative BOAS index determined, underwent a CT scan, routine BOAS surgery and finally post‐operative BOAS index determination in 6 to 8 weeks. The surgical procedures conducted were determined by the lesions identified through visual inspection, CT scans and rhinoscopy. These procedures may include a combination of folded flap palatoplasty, tonsillectomy, laryngeal ventriculectomy, laryngeal cuneiformectomy, alavestibuloplasty, radiofrequency turbinate tissue volume reduction and/or laser‐assisted turbinectomy. All surgeries were performed by board‐certified specialists experienced in airway surgery or by surgery residents under supervision. In this study, BOAS‐affected dogs that had a post‐operative BOAS index of greater than or equal to 50% (i.e. still clinically affected) were considered as those that had a poor surgical outcome. Those that had a good surgical outcome were considered to have a post‐operative BOAS index of less than 50%.

**FIG 1:**
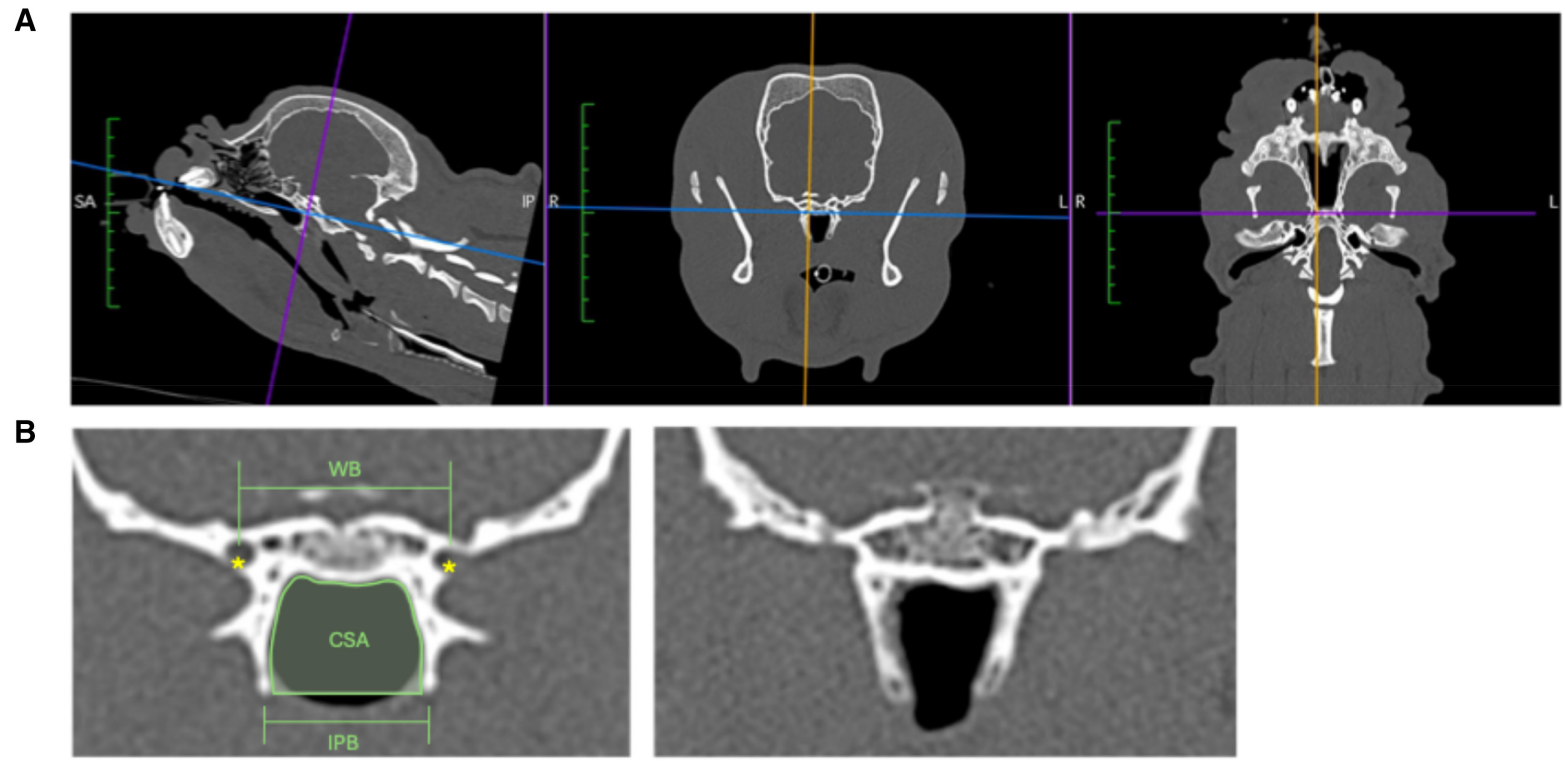
(A) Multiplanar reconstruction to obtain measurements at the level of the alar canals. (B; left) Demonstration of measurements obtained in the transverse plane at the level where the alar canals (*) were just visible. (B; right) Example of pterygoid bone medialisation in a French Bulldog. WB width of the basisphenoid bone, IPB Interpterygoid distance, CSA Cross‐sectional area of the nasopharynx.

Images of the head were acquired on a 16‐slice Aquilon CT with a slice thickness of 0.5 mm, with an image reconstruction index of 0.3, a pitch factor of 0.938 and a standard 512 × 512 matrix. Tube rotation time was 0.5 seconds and KVp = 100, mAs = 150. The images were acquired in bone and soft tissue windows [Bone: window width 3500 Hounsfield unit (HU), window level 1500 HU, soft tissue window width 150HU and window level 50HU]. Images were analysed using the software Horos (version 4.0, http://horosproject.org/, Nimble Co LLC d/b/a Purview in Annapolis, MD, USA). Multiplanar reconstructions were obtained at the level of the pterygoid hamuli. Landmarks used were the caudal edge of the pterygoid bone at the level where the alar canals were visible, the midline of the skull in line with the nasal septum and the horizontal edge of the basisphenoid bone (Fig 1A).

### Deriving the pterygoid medialisation index and cross‐sectional area of the nasopharynx

Measurements of the dimension of the pterygoid bones and the surrounding bony structures were performed using a bone window. Three measurements were obtained on the transverse plane at the level where the alar canals were just visible: width of the basisphenoid bone (WB), interpterygoid distance (IPB) and cross‐sectional area of the nasopharynx (CSA) following methods described by Kuzucu et al. (2020). The WB was the distance between the alar canals of the basisphenoid bone, and IPB was defined as the distance between the pterygoid hamuli. The CSA was the area of the bony frame that encases the nasopharynx. Figure 1B illustrates the method of obtaining the measurement. The value measured for the CSA was normalised with calculated body surface area (BSA) using the formula: BSA (m^2^) = 0.101 × (body weight in kg)^2/3^.

The ratio between WB and IPB was calculated. This ratio was denoted as the “medialisation index” as it quantified the extent of the pterygoid bone medialisation. This meant that the higher the medialisation index, the more medialised the pterygoid bones were. All measurements were performed by one of the authors (S. L. Y.) under the supervision of a Board‐certified European Specialist in Veterinary Diagnostic Imaging (M. A. G.). The evaluator was blinded to the patient's signalment and BOAS assessment results while performing the CT measurement. Measurements of the de‐identified study subjects were taken on Day 1 of obtaining CT slices and further repeated in a randomised order the following week.

### Statistical analyses

All statistical analyses were performed using R^6^ (version 4.3.0 for Mac). The intra‐class correlation coefficient (ICC) for each measurement obtained was calculated using the R's “irr” package to evaluate the intra‐observer reliability. The criteria used to assess inter‐observer ICC values were as follows:

Excellent intra‐observer repeatability: ICC greater than 0.9
Good intra‐observer repeatability: ICC between 0.75 and 0.9
Moderate intra‐observer repeatability: ICC between 0.5 and 0.75
Poor intra‐observer repeatability: ICC less than 0.5

The Welch two‐sample *t*‐test was used to compare the pterygoid bone conformation between brachycephalic and non‐brachycephalic dogs. To assess the normality of the data, the Shapiro–Wilk test was conducted, and the results were visually inspected using Q‐Q plots. Pearson's correlations were used to examine the relationship between pterygoid bone medialisation index and the CSA of the nasopharynx in each breed. Welch two‐sample *t*‐tests were used to identify the relationship between the medialisation index and the post‐operative outcome (binary data). Pearson's correlations were used to compare numerical values between breeds. Wilcoxon rank‐sum test as well as simple *t*‐tests were used to compare categorical variables between breeds. The significance level was set at 0.05 for all tests, unless otherwise stated.

## RESULTS

### Study population

A total of 144 BOAS‐affected dogs matched the inclusion criteria (47 Pugs, 64 French Bulldogs and 33 English Bulldogs) and 30 controls were included. Subject signalment is shown in Table 1.

**Table 1:** Signalment of the study subjects

	Pugs	French Bulldogs	Bulldogs	Controls
	(*n* = 47)	(*n* = 64)	(*n* = 33)	(*n* = 30)
Sex [female; *n* (%)]	19; 38%	18; 28%	7; 21%	10; 33%
Age [months; median (range)]	36; [10 to 126]	30; [4 to 98]	22; [4 to 75]	120; [8 to 150]
Body weight [kg; median (range)]	8.1; [5.5 to 14.8]	12.3; [7.2 to 16.4]	23.9; [13 to 35.5]	16.5; [3.9 to 39.4]

#### Intra‐observer repeatability of measurements

Intra‐observer repeatabilities of the three measurements (WB, IPB and CSA) ranged from good to excellent (ICC 0.882 to 0.998; all P values <0.001) (Table 2).

**Table 2:** Intra‐observer repeatability of each measurement

Measurement	ICC (95% CI)
WB	0.882 (0.841 to 0.912)
IPB	0.916 (0.886 to 0.938)
CSA	0.998 (0.997 to 0.999)

WB Width of basisphenoid bone, IPB Interpterygoid distance, CSA Cross‐sectional area, ICC Interclass correlation, CI Confidence interval

All P values were <0.0001

#### Pterygoid bone conformation in brachycephalic and non‐brachycephalic dogs

**FIG 2:**
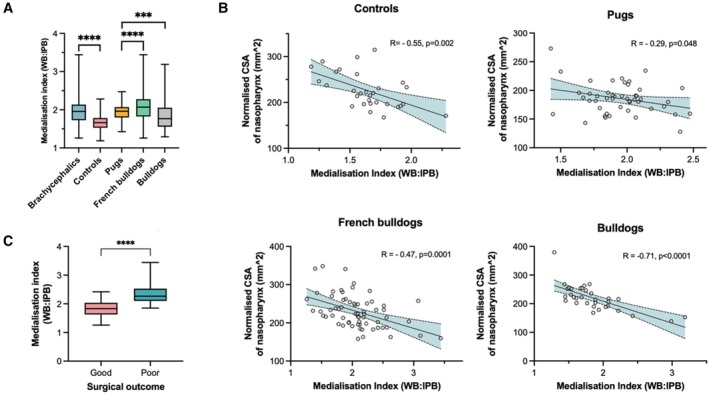
(A) Medialisation index compared between brachycephalic breeds and non‐brachycephalic controls. (B) The relationship between the normalised cross‐sectional area of the nasopharynx compared with the medialisation index in each brachycephalic breed and controls. (C) shows how French Bulldogs with a higher medialisation index have a poorer post‐surgical outcome compared to those with lower medialisation indices. WB Width of the basisphenoid bone, IPB Interpterygoid distance, CSA Cross‐sectional area of the nasopharynx. ***P < 0.001; ****P < 0.001.

The mean value for the medialisation index for the brachycephalic dogs group was significantly higher (mean = 1.982 ± 0.379) than that of control dogs (mean = 1.646 ± 0.239, P < 0.001) (Fig 2A). Within brachycephalic breeds, the medialisation index in Pugs (mean = 1.948 ± 0.240) was lower than that of French Bulldogs (mean = 2.072 ± 0.423) but higher than that of Bulldogs (mean = 1.853 ± 0.421). Both French Bulldogs (0.179) and Bulldogs (0.170) had high variance in medialisation index when compared to controls (0.057) and Pugs (0.057) (Fig 2A).

#### Relationship between pterygoid medialisation and cross‐sectional area of the nasopharynx

All three brachycephalic breeds and the controls showed a negative relationship between the medialisation index and the normalised CSA of the nasopharynx (Fig 2B). The Bulldog, in particular, had a strong negative correlation with R coefficient = −0.71 (P < 0.001) (Fig 2B).

#### Relationship between the medialisation index and post‐operative prognosis

Fifty‐seven per cent of brachycephalic dogs had a good surgical outcome while 43% had a poor surgical outcome. [Correction added on 19 September 2025, after first online publication: The percentage values in the preceding sentence were corrected.] The French Bulldogs with a poor surgical outcome (30/64) had a significantly greater pterygoid medialisation index (mean = 2.366 ± 0.327) when compared to dogs with a good surgical outcome (34/64, mean = 1.813 ± 0.271, P < 0.001) (Fig 2C). However, the pterygoid medialisation index did not differ between dogs with poor and good surgical outcomes in Pugs (P = 0.991) and Bulldogs (P = 0.655).

## DISCUSSION

The BOAS is characterised by various soft tissue and cartilaginous lesions, which have been extensively documented in the literature. This study reveals that medialisation of the pterygoid bones is more severe in brachycephalic breeds than in controls and can significantly contribute to nasopharyngeal narrowing. Importantly, this anatomical alteration is particularly associated with poor surgical outcomes in French Bulldogs.

Nasopharyngeal narrowing in BOAS‐affected dogs can be attributed to multiple anatomical structures. The rostral part of the nasopharynx is encased laterally by the palatine bones, ventrally by the hard and soft palate, and dorsally by the vomer and basisphenoid bone. The middle portions of the nasopharynx are bordered by the pterygoid bones laterally, basisphenoid bone dorsally and soft palate ventrally. The caudal portions of the nasopharynx are surrounded laterally by the medial pterygoid muscles and pharyngeal walls, and ventrally by the soft palate. Thickening of the soft palate and an oversized tongue can contribute to dorsoventral narrowing of the mid‐caudal nasopharynx. The bony structures are crucial in maintaining nasopharyngeal dimensions; any reduction in the bony encasement of the nasopharynx can potentially lead to fixed‐type airway restriction, as opposed to the more dynamic airway restrictions that occur further caudally.

Craniosynostosis is a known contributor to brachycephalia. This condition, defined by the early fusion of one or more cranial sutures, can significantly affect craniofacial morphology, including deviations in the position of the pterygoid bones (Kajdic et al., 2018). Specifically, the medial deviation of the pterygoid bones is thought to result from restricted growth and compensatory remodelling of the cranial base and adjacent structures. Similar patterns have been observed in syndromic craniosynostoses in humans, such as Crouzon and Apert syndromes, where complex suture fusions disrupt the normal development of the cranial vault, midface and skull base (Kajdic et al., 2018; Lu et al., 2021). The resulting medial orientation of the pterygoid plates has been linked to physiological consequences, such as narrower nasopharyngeal and pharyngeal airways, which impair respiratory function. In the present study, while the degree of pterygoid bone medialisation was higher in brachycephalic dogs than in control breeds, considerable overlap was observed. French Bulldogs and Bulldogs exhibited large variation in this metric, consistent with the findings in another larger scale study demonstrating a higher degree of craniofacial variation in French Bulldogs and Bulldogs compared with Pugs (Liu et al., 2017). The underlying factors driving medial deviation of the pterygoid bones within breeds remain unclear and will need to be further investigated.

Despite congenital malformation of the skull base, acquired forces may also contribute to the medial deviation of the pterygoid bones. The pterygoid processes are thin bony extensions that frame the lateral aspect of the rostral‐middle portion of the nasopharynx. In humans, particularly the caudal extremity of pterygoid processes known as the hamuli, can undergo shape alterations due to forces such as muscle tension and pulling (Katori et al., 2011; Krmpotić‐Nemanić et al., 2006; Oz et al., 2016; Putz & Kroyer, 1999). Chronic nasal obstruction, particularly in growing individuals, has long been associated with changes in craniofacial growth patterns, emphasising the potential influence of airway mechanics on the skull base anatomy (Shapiro, 1988). In brachycephalic dogs, nasal obstruction results from several structural abnormalities, including stenotic nares, narrowed nasal vestibules, aberrant and hypertrophied nasal conchae, narrowed choanae and hypertrophy of lateral nasal glands (Packer & Tivers, 2015). These obstructions significantly reduce the cross‐sectional area of the airway, with a 50% reduction, for instance, theoretically increasing negative airway pressure approximately fivefold under typical laminar flow conditions, according to Poiseuille's law. Turbulent airflow, often caused by aberrant nasal turbinates, introduces additional resistance not predicted by laminar flow models, compounding negative pressure effects. We speculate that the deformation of the pterygoid bones in brachycephalic dogs could be associated with increased negative pressure within the nasopharyngeal airway, potentially acting as both a driving force and consequence of airway obstruction over time. However, further experimental studies are required to investigate and confirm this association.

Given the complexity and multifactorial nature of lesions contributing to BOAS, conventional surgical interventions have limitations in fully alleviating the respiratory and other clinically associated signs of the condition. There are currently no effective surgical options reported to address the deformities of the pterygoid bones observed in BOAS‐affected dogs. Medialisation of the pterygoid bones can be easily identified via CT scans; alternatively, palpation of the pterygoid hamuli intra‐operatively from the oral cavity is also feasible. The assessment can help clinicians provide better‐informed predictions regarding post‐operative outcomes.

This retrospective study has several limitations. Firstly, the small number of Bulldogs available during the study period may have predisposed to type II errors, reducing the ability to detect potential associations. In addition, the relationship between the medialisation index and post‐surgical outcome could not be fully explored, as the sample size limited the statistical power. Further studies with larger cohorts could provide greater insight into this relationship. The small sample size and retrospective nature of the study contributed to the statistically significant result when investigating the relationship between age and body weight between brachycephalic dogs and controls. The median age of brachycephalic dogs in this study was 3 years or under, whereas that of the controls had a median of 10 years. This was mainly due to the samples used for controls where patients often obtaining CT scans were involved in the staging of neoplastic disease. Secondly, the medialisation index was quantified using a single slice obtained from the transverse plane of the CT scan, which did not evaluate or represent the entirety of the bony structure forming the nasopharynx. Comprehensive three‐dimensional imaging analyses may yield more accurate and holistic assessments in future research. Finally, this study investigated post‐operative outcome over a short‐ to medium‐term follow up period. A longer‐term longitudinal study would be beneficial to better determine the true impact of surgical intervention on long‐term outcome in these patients.

In conclusion, pterygoid bone malformation is a notable feature in brachycephalic dogs. Among brachycephalic breeds, French Bulldogs with a medialised pterygoid bone conformation experience worse post‐operative outcomes after standard BOAS surgery. This highlights the importance of evaluating the extent of nasopharyngeal narrowing caused by bony structures in predicting surgical success and post‐operative prognosis. Moreover, incorporating the medialisation index into breeding programmes could play a pivotal role in improving the welfare of future generations of French Bulldogs by reducing extreme brachycephalic features that compromise health and quality of life.

## Author contributions

S. L. Yuen: Data curation (equal); formal analysis (lead); funding acquisition (equal); investigation (equal); methodology (equal); project administration (equal); validation (equal); visualisation (equal); writing – original draft; conceptualisation (equal). M. A. Genain: Methodology (equal); funding acquisition (equal); writing – review and editing (equal); project administration (equal); validation (equal); visualisation (equal); conceptualisation (equal); supervision (equal). J. F. Ladlow: Data curation (equal); writing – review and editing (equal). N.‐C. Liu: Data curation (equal); conceptualisation (equal);formal analysis (equal); funding acquisition (equal); investigation (equal); methodology (equal); supervision (equal); project administration (equal); validation (equal); visualisation (equal); writing – review and editing (equal).

## Conflict of interest

None of the authors of this article has a financial or personal relationship with other people or organisations that could inappropriately influence or bias the content of the paper.

## Data Availability

The data that support the findings of this study are available from the corresponding author upon reasonable request.
